# Cell-controlled dynamic surfaces for skeletal stem cell growth and differentiation

**DOI:** 10.1038/s41598-022-12057-z

**Published:** 2022-05-17

**Authors:** Hilary J. Anderson, Jugal Kishore Sahoo, Julia Wells, Sebastiaan van Nuffel, Hala S. Dhowre, Richard O. C. Oreffo, Mischa Zelzer, Rein V. Ulijn, Matthew J. Dalby

**Affiliations:** 1 Centre for the Cellular Microenvironment, Institute of Molecular, Cell & Systems Biology, MVLS, University of Glasgow, Joseph Black Building, Glasgow, G12 8QQ UK; 2 Department of Pure and Applied Chemistry, Technology and Innovation Centre, University of Strathclyde, Glasgow, G1 1RD UK; 3 Bone and Joint Research Group, Centre for Human Development, Stem Cells and Regeneration, Institute of Developmental Sciences, University of Southampton, Southampton, SO16 6YD UK; 4 School of Pharmacy, Biodiscovery Institute, University Park, University of Nottingham, Nottingham, NG7 2RD UK; 5 Nanoscience Initiative at Advanced Science Research Center (ASRC) of the Graduate Center of the City University of New York, New York, USA; 6 Department of Chemistry Hunter College, City University of New York, New York, USA; 7 Ph.D. Programs in Biochemistry and Chemistry, The Graduate Center of the City University of New York, New York, USA; 8 Present Address: Department of Biomedical Engineering, Science and Technology Centre, Tufts University, 4 Colby St., Medford, MA 02155 USA; 9 Present Address: M4I, Faculty of Science and Engineering, Maastricht University, Maastricht, The Netherlands; 10 Present Address: Department of Ophthalmology, Stanford University School of Medicine, Stanford, CA 94305 USA

**Keywords:** Biomaterials, Bioinspired materials, Biomaterials - cells, Mesenchymal stem cells, Biomimetics

## Abstract

Skeletal stem cells (SSCs, or mesenchymal stromal cells typically referred to as mesenchymal stem cells from the bone marrow) are a dynamic progenitor population that can enter quiescence, self-renew or differentiate depending on regenerative demand and cues from their niche environment. However, ex vivo, in culture, they are grown typically on hard polystyrene surfaces, and this leads to rapid loss of the SSC phenotype. While materials are being developed that can control SSC growth and differentiation, very few examples of dynamic interfaces that reflect the plastic nature of the stem cells have, to date, been developed. Achieving such interfaces is challenging because of competing needs: growing SSCs require lower cell adhesion and intracellular tension while differentiation to, for example, bone-forming osteoblasts requires increased adhesion and intracellular tension. We previously reported a dynamic interface where the cell adhesion tripeptide arginine-glycine-aspartic acid (RGD) was presented to the cells upon activation by user-added elastase that cleaved a bulky blocking group hiding RGD from the cells. This allowed for a growth phase while the blocking group was in place and the cells could only form smaller adhesions, followed by an osteoblast differentiation phase that was induced after elastase was added which triggered exposure of RGD and subsequent cell adhesion and contraction. Here, we aimed to develop an autonomous system where the surface is activated according to the need of the cell by using matrix metalloprotease (MMP) cleavable peptide sequences to remove the blocking group with the hypothesis that the SSCs would produce higher levels of MMP as the cells reached confluence. The current studies demonstrate that SSCs produce active MMP-2 that can cleave functional groups on a surface. We also demonstrate that SSCs can grow on the uncleaved surface and, with time, produce osteogenic marker proteins on the MMP-responsive surface. These studies demonstrate the concept for cell-controlled surfaces that can modulate adhesion and phenotype with significant implications for stem cell phenotype modulation.

## Introduction

Skeletal stem cells (SSCs, also referred to as mesenchymal stromal cells or mesenchymal stem cells, MSCs) are dynamic cells that can undergo expansion through self-renewal and differentiation along the adipogenic, chondrogenic, reticular and osteogenic lineages^1^. MSCs have been stated to be available from several locations including bone marrow, adipose tissue and olfactory mucosa^2,3^; the best characterised being the bone marrow SSCs. In their niches, SSCs rely on information from the extracellular matrix and other cells in order to remain quiescent to protect them from DNA damage, self-renew to expand the SSC population or differentiate to drive regeneration^4^. In vitro, typically grown on hard polystyrene, the information SSCs rely on to self-renew is absent and consequently, SSCs rapidly differentiate, lose their phenotype and express a fibroblast phenotype^5,6^.

Surface strategies to maintain SSC phenotype are emerging, for example, nanotopograhical patterns that promote SSC growth without loss of phenotype^6^. These surfaces act to reduce SSC adhesion formation compared to planar surfaces resulting in changes in mitogen related signalling (e.g., extracellular signal-related kinase, ERK1/2, and c-jun n-terminal kinase, Jnk)^7–9^. Integrins, transmembrane receptors that form cell adhesions and that ligate to peptide motifs in the extracellular matrix, such as RGD, link to signalling proteins such as focal adhesion kinase, FAK, and the actin cytoskeleton to effect phenotype-linked signalling pathways^10–12^. These mitogens are well known for their roles in SSC differentiation along with Rho A kinase signalling to facilitate adipogenesis where lack of spreading results in low levels of mitogen stimulation and lack of intracellular tension or to drive osteogenesis through mitogen activation of transcription factors such as runt related transcription factor 2 (RUNX2) and adhesion-driven spreading providing high levels of intracellular tension^13–15^.

If it is assumed SSCs have cytoskeletal tension-related phenotypes, adipocytes represent very low tension, fibroblasts intermediate tension and osteoblasts high-tension. It is emerging that SSC self-renewal occurs with slightly reduced tension from the fibroblast phenotype^6,9^. This is sensible as SSCs have similar morphology to fibroblasts as opposed to adipocytes or osteoblasts, originally being defined as fibroblast colony-forming units^16^. In fact, the morphology-tension difference between a fibroblast and the SSC in culture is modest and yet has a significant impact between growing fibroblasts and a major regenerative stem cell type^6,9^. To maintain the SSC phenotype, the mitogens appear to regulate the cell cycle in a manner that reduces the SSC ability to differentiate. For example, phosphorylated retinoblastoma protein, a negative regulator of cell cycle associated with RUNX2 activation, is down-regulated compared to differentiating SSCs and cyclin dependant kinase 6, a positive regulator of cell cycle that reduces cellular BMP-2 (bone morphogenetic protein) sensitivity, is up-regulated in self-renewing SSCs^8,17,18^.

As reported by Stein and Lian in 1993, as well as contemporary studies, growth is reduced to allow for SSC differentiation^19,20^. This means that differentiated progeny, such as adipocytes and osteoblasts tend to be slow-growing, while self-renewing SSCs proliferate rapidly, like fibroblasts^8,9^. However, it is these differentiated progenies that are required in large numbers to enable clinical therapy. Bone, for example, is the second most transplanted tissue after blood and bone graft is in short supply^21^. Graft can be harvested from donor sites as autograft, but the amount is limited and there is associated donor site pain and morbidity^22,23^. Allograft is necessarily decellularised and so lacks ‘the biology’ (i.e. the cells) needed to affect efficient repair^21^. Tissue engineering using osteoblasts represents a future vision, where lab-grown osteoblasts are cultured on an osteogenic scaffold material with appropriate biological and biomechanical cues^24–26^.

Thus, there is a need to move away from using plastic cultureware designed for the growth of fastidious cell lines^27^ to strategies designed specifically for stem cell populations to facilitate cell growth and differentiation. These strategies may take the form of 3D gels or microbeads^27^, but here, we will focus on a novel surface functionalisation strategy. Specifically, we will focus on a strategy designed to promote, initially, SSC self-renewal, cell number expansion, and subsequently bone-specific differentiation to produce a regenerative cell type where there is clinical need. Furthermore, a central goal is the development of an autonomous, cell-controlled platform, rather than a user-controlled, manner.

A growing number of studies have identified dynamic materials for SSC culture. For example, photoactive hydrogels illustrate that while growth control remains poor in such systems, there is potential for targeting osteogenesis from SSCs over short culture times^28^. Electro-active surfaces^29–31^ and protein responsive materials^32^ are also reported. However, these studies rely on non-biocompatible chemistries and the application of conducting materials/electrochemical potentials that may affect cell response. Degradation^33^ and stress relaxation^34^ of viscoelastic hydrogels also form further strategies that can dynamically tune the SSC phenotype; however, these properties can be hard to control. We have adapted a surface-based strategy that has previously been described to use enzymatic activation to provide a natural stimulus under user-control to trigger changes in material properties, with advantages of biocompatibility and selectivity^35^. This surface approach was based on solid-phase peptide synthesis (SPPS) directly onto the solid surface, which was used to create an enzyme cleavable sequence to reveal the cell-adhesive tripeptide, RGD, to SSCs. The sequence A↓ARGD (arrow denotes elastase cleavage site) was capped with the bulky blocking group Fmoc (fluorenylmethyloxycarbonyl) shown to prevent sterically access to the RGD underneath (Todd et al.,^36^https://pubs.rsc.org/en/content/articlelanding/2007/sm/b618256a/unauth) and subsequently with PEG [ref] and covalently bound to glass substrates using a polyethylene glycol (PEG) linker. It was noted that with Fmoc (or PEG) in place, the SSCs produced smaller adhesions, generated less myosin-induced intracellular tension, and retained markers of stem cell phenotype. However, when the blocking group was cleaved with elastase, the SSCs produced osteoblast-specific super-mature adhesions (cell adhesions > 5 µm long)^37^, increased myosin phosphorylation and differentiated along the osteoblast lineage^38^.

**Figure 1:**
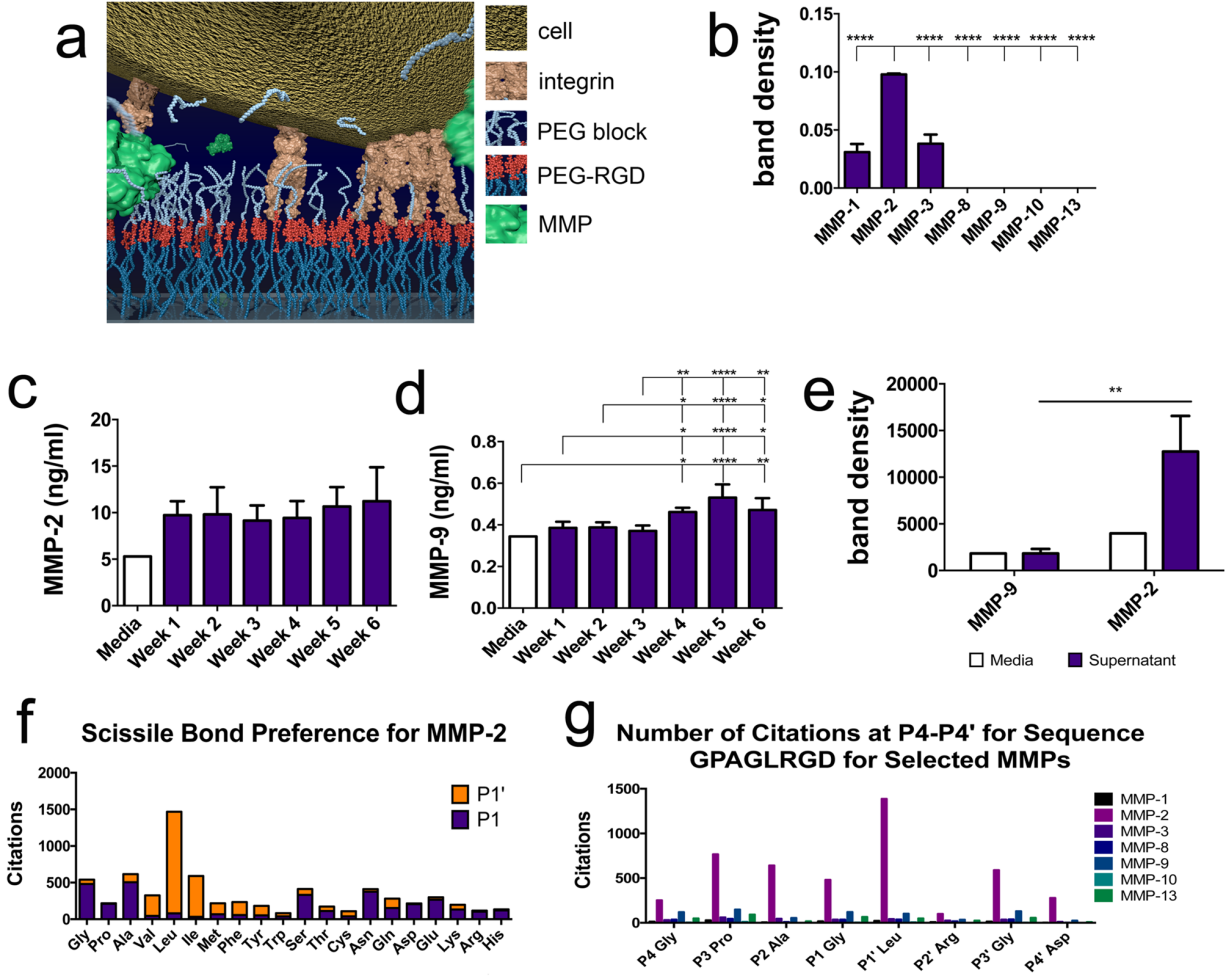
Selecting MMPs and cleavage sequence. (**a**) Schematic of the SSC cleavable surface concept with MMP-driven removal of PEG revealing RGD to the cells. (**b**) After 3 weeks of culture, MMP protein array was performed by densitometry measurements of the array membranes demonstrating high levels of MMP-2 expression (n = 3). (**c,d**) ELISA showed constant expression of MMP-2 and increasing expression of MMP-9 with time (n = 3). (**e**) Zymography was used to show that only MMP-2 was expressed by the SSCs in active form after 3 weeks of culture (n = 3). (**f**) MERPOS data gave consensus of an 8-amino acid sequence with a strong preference for L at P1′; allowing us to design a GPAG↓LRGD sequence for MMP-2 cleavage that would leave RGD on the coverslip. (**g**) GPAG↓LRGD sequence is highly cited as being MMP responsive For (**b–e**), results are mean ± SD, statistics by ANOVA and Tukey test where *p < 0.05, **p < 0.01, ***p < 0.001 and ****p < 0.0001. If stars are not shown on the graphs in (**b–e**), it denotes no significant difference was observed between the treatment and relevant control.

The current studies detail the development of a cell-controlled surface, where AA is replaced by a peptide linker sensitive to matrix metalloprotease (MMP) action. The hypothesis under test was that accumulation of MMPs, such as MMP-2 typically expressed by SSCs as the cells approach confluence^39^, could cleave the blocking group to expose RGD (Fig. 1a). We note that in the previous work^38^ the Fmoc blocking group could become biofouled, preventing AA cleavage and so the antifouling PEG group has been used as a blocker.

### SSC MMP profile and design of the surface

Following the culture of SSCs (Stro-1^+ve^ adherent cells from human bone marrow with stem cell characteristics shown by both enriched CFU-F formation and in vivo bone formation^40,41^) on tissue culture plastic, the cell supernatant was collected for MMP analysis. First, an MMP protein array was used after 3 weeks of culture to show that MMP-2 was expressed in abundance (Fig. 1b). MMP-2 is collagenase that can be expressed by SSCs^39,42^. We note that MMP-9 expression is also noted in SSCs in the literature^39,42^ and so both MMP-2 and MMP-9 expression was quantified by ELISA over a 6-week time-course. MMP-2 was expressed constantly (Fig. 1c) while MMP-9 expression was noted with time (Fig. 1d). It is important to note that MMPs can be expressed in both inactive and active forms and thus zymography was used to quantify MMP-2 and 9 activity after 3 weeks of culture. Results showed only MMP-2 was significantly expressed in an active form compared to that found in cell culture media (Fig. 1e). Thus, as MMP-2 is expressed by the SSCs in active form, we decided to design a cleavage sequence that was responsive to MMP-2 activity.

To define the peptide sequence of the scissile bond we used the MEROPS database^43^ that indicated that the MMP-2 sequence preference consisted of an 8-amino acid peptide sequence (P4–P4′). For the P1 and P1′ cleavage site, the database indicated a preference for small amino acids with G or A equally likely at P1, however, literature reports consistently state that L is favoured at P1′ (1387 citations) (Supplementary Fig. S1, Fig. 1f). We thus designed a peptide cleavage sequence with RGD incorporated at the prime side that would remain on the coverslip post-MMP activity; the final GPAG↓LRGD sequence has been cited almost 1500 times as being MMP2 sensitive (Fig. 1g). Coverslips were fabricated to present PEG-GPAG↓LRGD-PEG-glass to the cells (surfaces with this complete sequence are denoted as DIGE-D for digestible with RGD).

**Figure 2:**
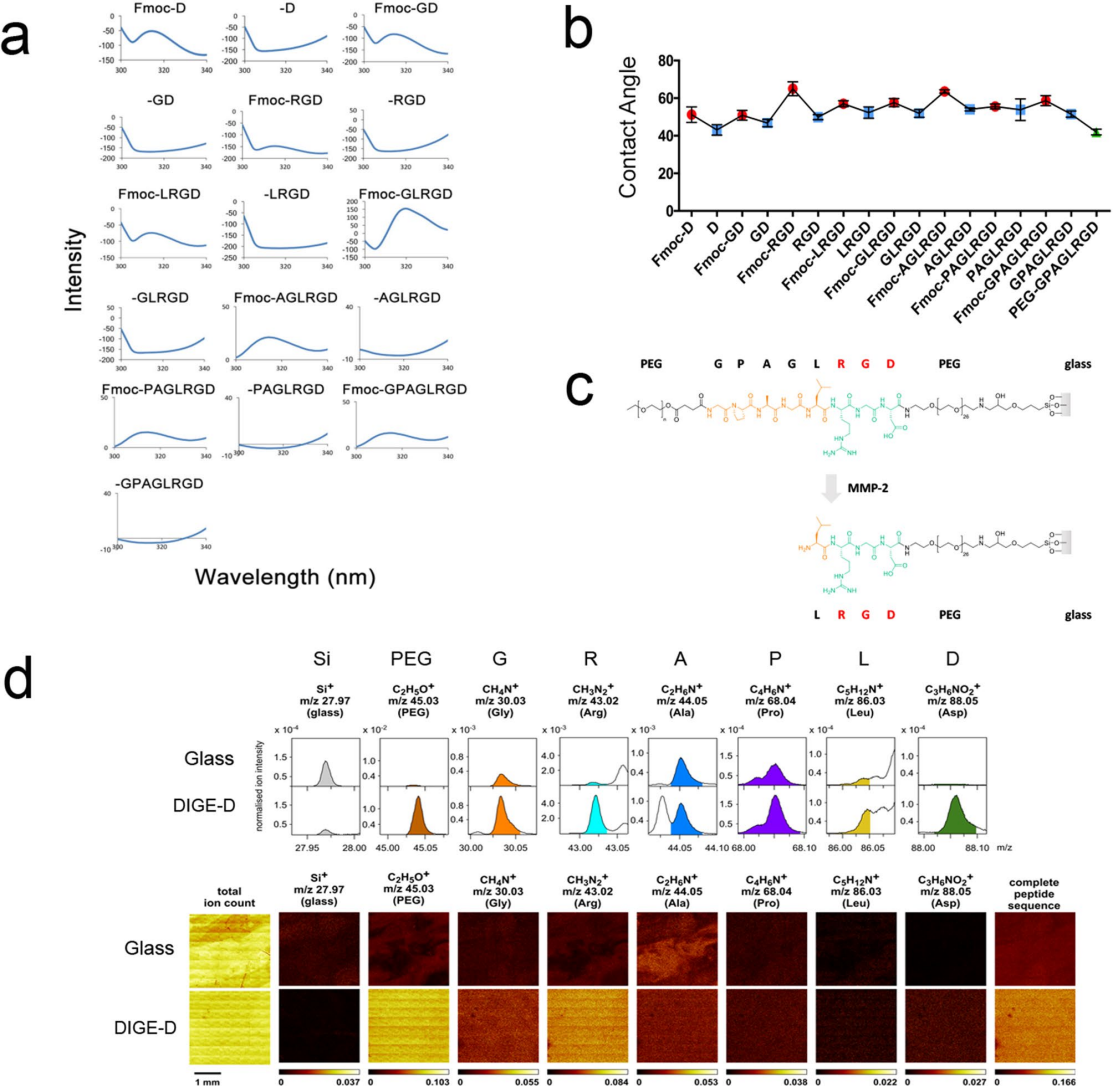
Characterisation of solid-phase peptide synthesis (SPPS) dynamic surface. (**a**) Surface fluorescence spectroscopy following addition/removal of the Fmoc group (Fmoc peak seen at ~ 315 nm) during synthesis. (**b**) Contact angle measurement following addition (red)/removal (blue) of the Fmoc group during synthesis and the final PEG-capped peptide sequence (green) (graph shows mean ± SD, 50 images per dataset with 15 datasets taken across 3 substrates (n = 3)). (**c**) Scheme showing complete RGD surface with MMP cleavable group and PEG blocker before and after MMP cleavage. (**d**) Tof–SIMS data showing that expected amino acids (G, P, A, L, R and D) and PEG were present post-synthesis. Together, this data indicates complete PEG-GPAGLRGD synthesis.

To fabricate the cell culture surfaces, glass coverslips were cleaned, silanised and a PEG linker attached. The peptide sequence was built using Fmoc-protected amino acids grafted from the bottom-up and the complete sequence was hidden beneath a second PEG chain. Fluorescence spectroscopy, detecting the presence or absence of Fmoc^44^ (Fig. 2a), and water contact angle (WCA) measurements, again detecting the presence or absence of the hydrophobic Fmoc group (Fig. 2b) was used to follow the synthesis of the MMP-2 cleavable surface (Fig. 2c).

In addition to WCA and fluorescence spectroscopy, time of flight secondary ion mass spectrometry (TOF–SIMS) was used to detect the presence of the amino acids used to build the MMP-2 responsive peptide sequence on the coverslips (Fig. 2d). On the peptide surface, ions related to all six amino acids (Ala, Gly, Asp, Arg, Pro and Leu) and the PEG chain were detected. The intensity of the Si+ ion considerably decreases after peptide attachment, indicating that the sample surface has been modified. While some of the amino acid-related ions were also present on the control sample (clean glass), all ions but that associated with Ala (C_2_H_6_O^+^) showed a significant increase in intensity after peptide modification. Detection of the C_2_H_6_O^+^ ion on the control sample and the resulting lack in a distinct increase of the intensity of that ion on the peptide sample can be explained by the fact that the (C_2_H_6_O^+^) ion is a generic ion that can also originate from adventitious carbon contaminations. Amino acids and the PEG group were present after the synthesis (Fig. 2d). Moreover, the distribution of these ion intensities over a larger surface area is largely homogenous, demonstrating that the surface modification is reasonably uniform across the coverslip. Following our previously developed protocol^34^, the monitoring of sequential attachment of amino acids by fluorescence and WCA measurements and the confirmation of the overall surface chemistry by ToF–SIMS combined confirm the correct peptide sequence has been prepared on the surface.

**Figure 3:**
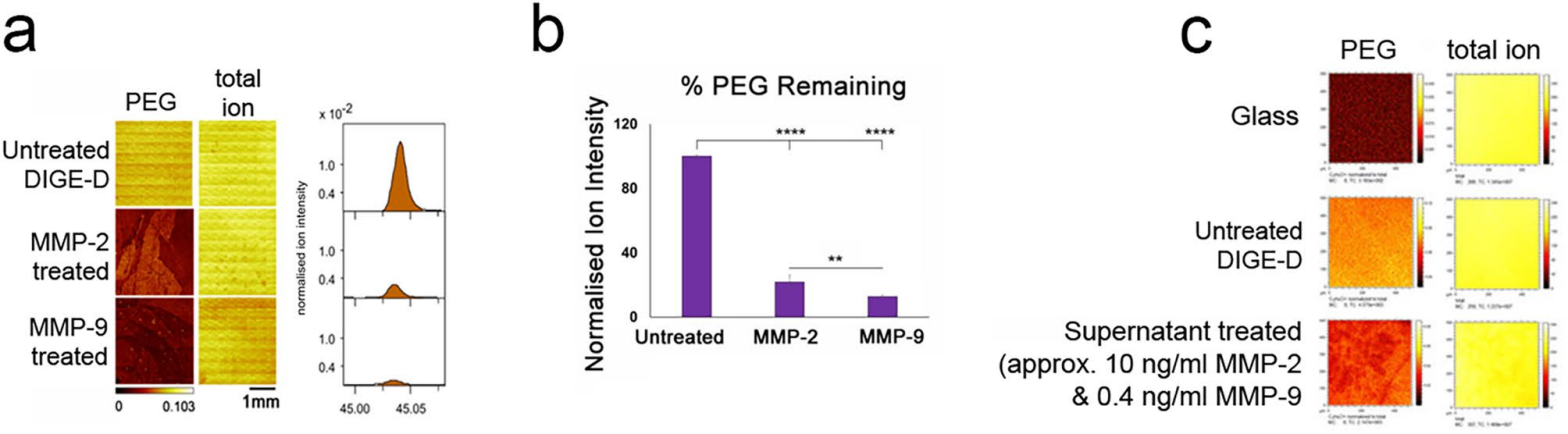
MMPs can cleave PEG from the peptide and reveal RGD. (**a,b**) Tof–SIMS data showing removal of the PEG group with the addition of MMPs-2 and 9 in more detail. (**c**) Treatment of the DIGE-D surface with SSC supernatant containing 10 ng/mL of MMP-2 (active) and 0.4 ng/mL of MMP-9 (latent) resulted in some cleavage of PEG.

Looking at the effect of adding serum-free media spiked with either MMP-2 or MMP-9 (20 ng/mL and 0.25 ng/mL respectively), noting the scissile bonds can be MMP promiscuous and still having an interest in the high levels of MMP-9, albeit inactive as seen in Fig. 1d, TOF–SIMS showed a reduction in the P and A amino acids as well as the capping PEG group compared when both MMP-2 and 9 were added to the full peptide sequence suggesting successful cleavage (§ cleaved compared to $ uncleaved) (Supplementary Fig. S2). Figure 3a,b show the removal of PEG by exogenous MMP-2 and MMP-9.

Figure 1c,d showed that ~ 10 ng/mL of endogenous MMP-2 and ~ 0.4 ng/mL of endogenous MMP-9 were expressed by the SSCs in culture. To test if these levels could cleave the scissile bond in the peptide sequence in serum-containing conditions (i.e., where competitor proteins may be present for the MMPs to act upon), media collected after 3 weeks of the culture of the SSCs on glass was added to the sequence before TOF–SIMS analysis. The supernatant treated surface could be seen to have reduced PEG signal intensity indicating successful PEG blocking group removal albeit at reduced efficiency compared to the use of exogenous MMPs in serum-free conditions (Fig. 3c). We note that we also see protein adsorption from this sample, so the quantification must be interpreted with some caution (Supplementary Fig. S3).

### SSC biocompatibility and adhesion

To understand the biocompatibility of the surface and cell adhesion to the surface, we performed some short-term experiments with a range of glass, RGD (high cell adhesion) and RGE (low cell adhesion) controls as shown in Table 1.

**Table 1:** Surfaces used for cell culture experiments.

Abbreviation	Sample
Glass	Glass coverslip
PEG-RGD	RGD attached to glass via PEG linker (glass-PEG-RGD)
LRGD	Pre-cleaved with exogenous 1 mg/mL MMP-2
DIGE-D	Full length, MMP-2 digestible, sequence (PEG-GPAG↓LRGD-PEG-glass)
PEG-RGE	RGE attached to glass via PEG linker (glass-PEG-RGD)
LRGE	Pre-cleaved with exogenous 1 mg/mL MMP-2
DIGE-E	Full length, MMP-2 digestible, sequence (PEG-GPAG↓LRGE-PEG-glass)

**Figure 4:**
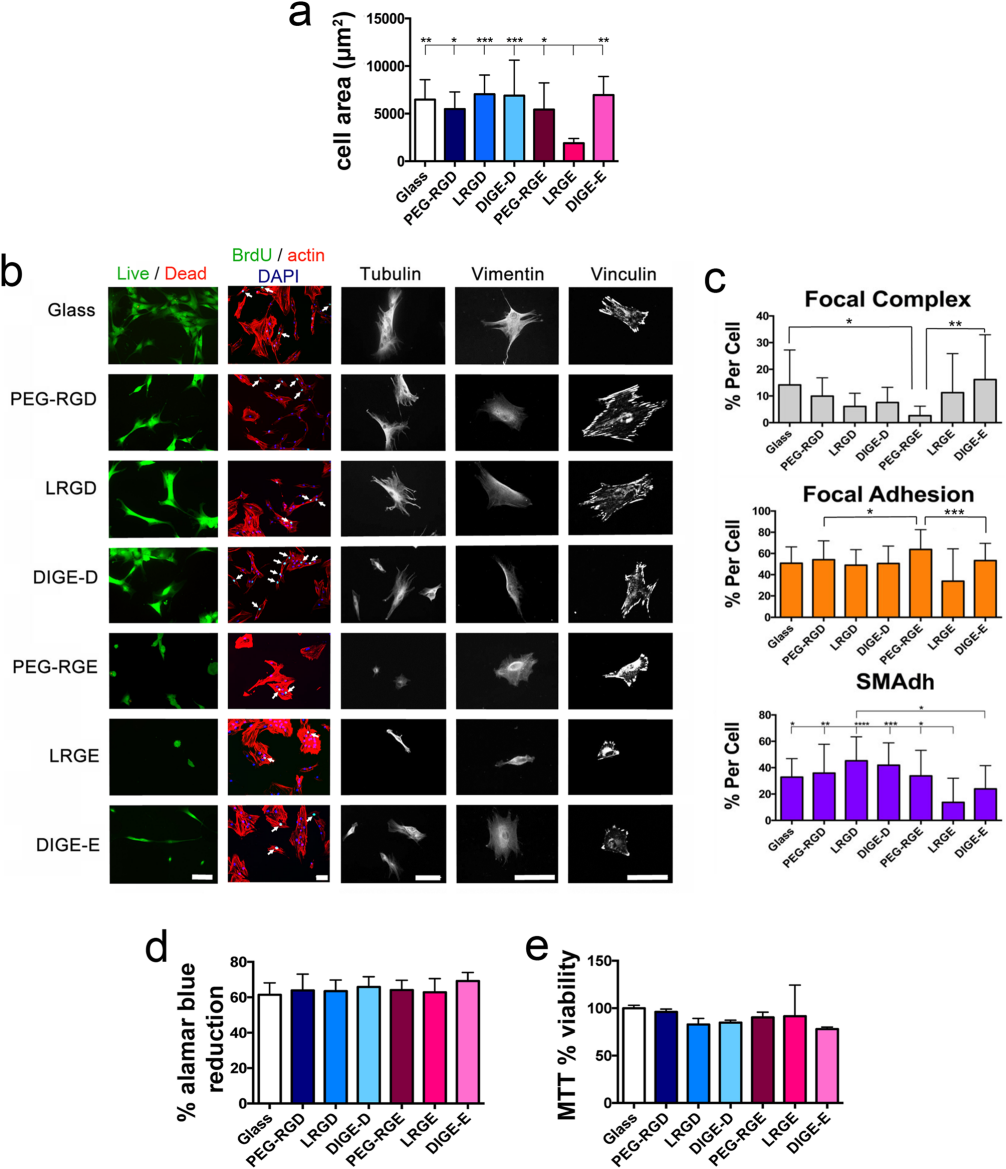
SSC biocompatibility. (**a**) Cell area measurements after 24 h of culture on the samples showing that RGD containing surfaces and DIGE-E supported similar cell spreading compared to glass while LRGE, where the RGE was exposed, reduced SSC spreading (n = 15). (**b**) Viability, proliferation and cytoskeletal analysis showed high cell viability on all surfaces. Growth and cytoskeletal organisation were reduced on the LRGE surface and PEG-RGE surface where the RGE group was exposed. (**c**) Focal adhesions were categorised as FC (< 2 µm), FA (2–5 µm) and SMAdh (> 5 µm) and expressed as % total adhesions (n = 15). The main difference to indicate cells responding to the PEG cleavage was seen in the SMAdhs, where LRGD and DIGE-D gave the largest differences to the RGE containing surfaces. (**d**) Alamar blue and MTT metabolic assays showing no differences (n = 3). Graphs show mean ± SD, statistics by ANOVA and Tukey test where *p < 0.05, **p < 0.01, ***p < 0.001 and ****p < 0.0001. If stars are not shown on the graphs, it denotes no significant difference was observed between the treatment and relevant control. Please note that SSCs purchased from Promocell GMBH rather than stro-1^+ve^ cells were used to create this figure.

It was observed that after 24 h of culture, SSCs were viable on all the surfaces, but SSCs displayed reduced cell spreading (measurement of cell area) on RGE containing surfaces reflecting the low adhesivity of the RGE group (Fig. 4a,b). After 24 h of SSC culture, we observed the cytoskeleton proteins tubulin and vimentin, involved in cell metabolic activity, and observed/measured focal adhesion size (Fig. 4b). Tubulin microtubules and vimentin intermediate filaments reflected the cell area data with a propensity for enhanced cell spreading displaying organised tubules and filaments on RGD containing surfaces. Vinculin staining revealed that cells could form adhesions on all the surfaces, including RGE containing surfaces (Fig. 4b,c). However, measurement of adhesion length and sub-classified as focal complex (small, transient adhesions < 2 µm long), focal adhesions (stable adhesions 2–5 µm long) and super-mature adhesions (very large adhesions required to support osteogenesis, > 5 µm long), indicated that the cleaved LRGD sample contained the most super-mature adhesions with RGE containing surfaces supporting the fewest super-mature adhesions per cell (Fig. 4c).

After 1 week of culture on the surfaces, alamar blue reduction and MTT assays were used to calculate cell metabolic activity as a measure of growth. There were no differences between surfaces implying similar cell numbers (Fig. 4d,e). Again, bromodeoxyuridine (BrdU) incorporation after 1 week showed S-phase cells present on all materials (Fig. 4b).

Taken together, this data suggests a cell adhesion lag phase on RGE containing surfaces illustrating that initial cell response is quite different depending on sample, but that the cells can spread and proliferate to levels comparable to cells on the glass control, with time.

### SSC phenotype

**Figure 5:**
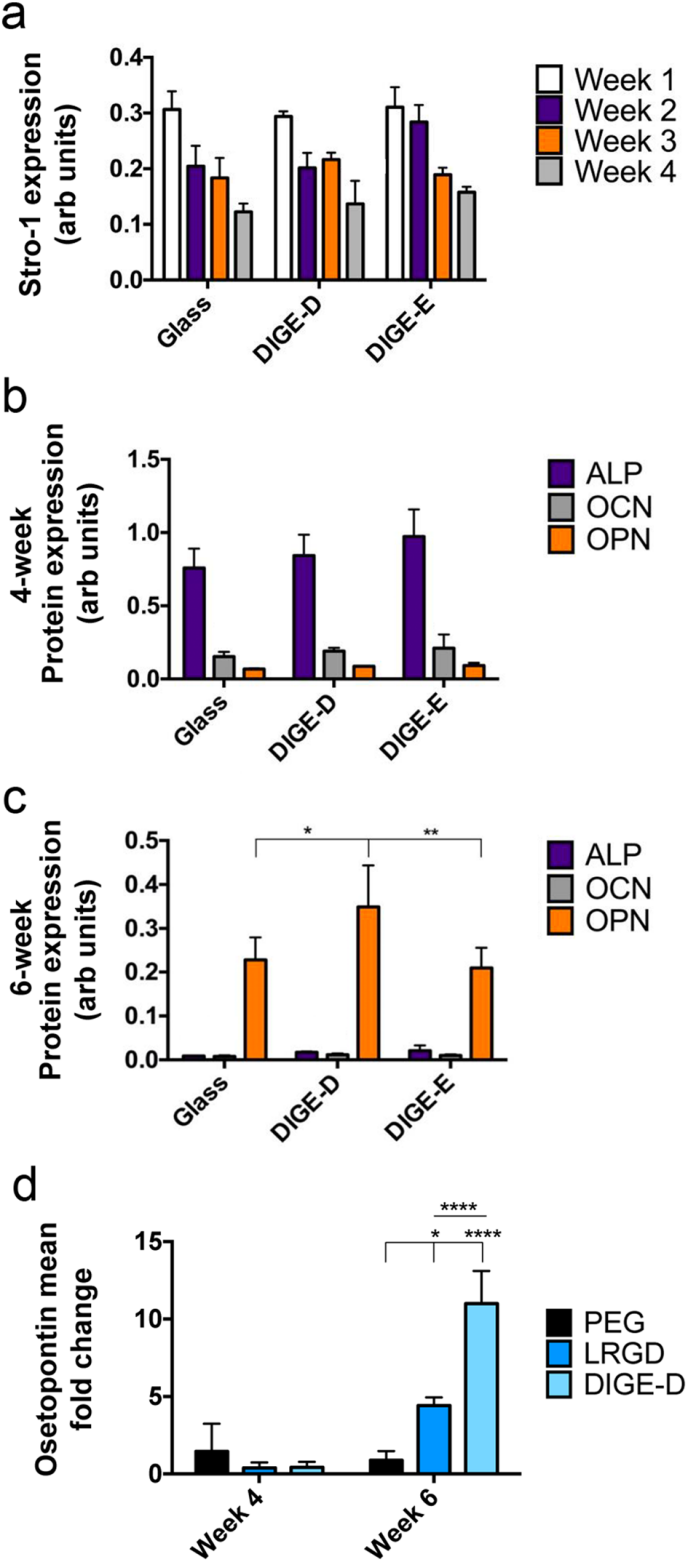
SSC phenotype on DIGE-D surface. (**a**) In cell western (ICW) data for Stro-1 showing no difference between SSCs cultured on glass control and DIGE-D and DIG-E surfaces showing that cells express similar levels of this SSC marker on the SPPS test and control surfaces when the RGD/RGE is hidden by the PEG blocking group. (**b,c**) ICW data for alkaline phosphatase (ALP), osteopontin (OPN) and osteocalcin (OCN) after 4 weeks (**b**) and 6 weeks (**c**) of culture. No differences were noted between samples at 4 weeks, but at 6 weeks, higher levels of OCN expression were noted in SSCs on the DIGE-D surface. (**d**) QPCR for OPN transcripts after 4 and 6 weeks of MSC culture on PEG, LRGD and DIGE-D surfaces. At 4 weeks no change was seen. However, at 6 weeks, the DIGE-D MMP-responsive surface supported significantly greater OPN expression. Graphs show mean ± SD, n = 3, statistics by ANOVA and Tukey test where *p < 0.05, **p < 0.01, ***p < 0.001 and ****p < 0.0001. If stars are not shown on the graphs, it denotes no significant difference was observed between the treatment and relevant control.

To study phenotype, SSCs were cultured on the full substrates (GPAG↓LRGD, DIGE-D, GPAG↓LRGE and DIG-E) for up to 6 weeks. First, we looked at Stro-1 expression, as a SSC marker, after 4 weeks of culture. In cell western analysis showed that Stro-1 expression reduced on all samples, glass control, DIGE-D and DIGE-E, similarly with time (Fig. 5a). This indicates that the DIGE-D sample does not encourage lineage commitment at early stages of culture and that the SSCs should respond to the osteogenic RGD^45^ when revealed.

SSCs were then examined for osteogenic marker proteins alkaline phosphatase (ALP), osteopontin (OPN) and osteocalcin (OCN)^19^ after 4 and 6 weeks of SSC culture on the surfaces. At 4 weeks, no difference was seen between any of the samples indicating no onset of osteogenesis (Fig. 5b). At 6 weeks, however, an increase in OPN was noted, importantly, only on the DIGE-D surface (Fig. 5c). This indicates potential osteogenesis on the MMP-2 cleavable, RGD containing sample over that observed on glass or RGE containing controls, where fibroblastic phenotypical drift is hypothesised.

Next, qPCR was used to examine for OPN expression in SSCs at 4 and 6 weeks cultured on the LRGD and MMP-2 responsive DIGE-D surfaces compared to PEG controls. It was observed again that at 4 weeks there was no expression of OPN. However, after 6 weeks of culture, significantly greater OPN expression was noted on DIGD-D compared to LRGD and PEG (Fig. 5d). This result agrees with data presented in Ref.^38^ that illustrates that when elastase is used to cleave the blocking group to reveal RGD, enhanced osteogenesis is noted compared to use of RGD alone; i.e. the reveal of RGD enhanced osteogenic commitment. Looking at marker transcripts for other lineages, it was noted that the DIGE-D surface is osteospecific (Supplementary Fig. S4).

### Summary

We have designed an MMP-responsive surface for SSC culture. Using exogenous, active, MMPs, we show that the PEG blocking group can be efficiently cleaved to reveal RGD. The current studies further show that MMPs released during SSC culture can cleave the surface, but much less efficiently. This is likely because the SSCs were observed to express low levels, ~ 10 ng and ~ 0.4 ng of MMP-2 and-9 respectively, with only MMP-2 in the active form resulting in incomplete removal of PEG.

When SSCs were cultured on the solid phase peptide synthesis (SPPS) fabricated surfaces, comparable viability, metabolic activity and growth was seen as for SSCs cultured on the glass coverslip control. Use of RGE rather than RGD resulted in reduced cell adhesion and spreading showing that RGD was present in the DIGE-D for cells to respond to; on RGE blocked with PEG, SSCs could adhere and grow. Expression of Stro-1 with time in culture showed a similar profile on DIGE-D to glass control. Thus, while SSC phenotype was not actively enhanced by the designed surface, the surface was not detrimental to SSC phenotype. Typically, under osteogenic conditions, SSCs that will differentiate along the osteoblastic lineage take approximately 3–4 weeks to express mature osteoblast markers such as osteopontin and osteocalcin^19,20^. In the current studies, approximately 6 weeks of culture was required to observe expression of these markers on the DIGE-D surface. This suggests a delayed expression as the cells switched the surface from ‘grow’ to ‘differentiate’.

We have produced a surface that permits SSC growth followed by SSC differentiation when the cells produce sufficient MMP-2. Clearly optimisation is required. We propose that the RGD groups need to be better hidden to enhance retention of SSC phenotype relative to glass during the growth phase. Subsequently, optimisation of cleavage is required to allow enough PEG to be cleaved to drive robust osteogenesis. Further, while low adhesion controls did reduce adhesion and spreading, cells could take hold and grow. These studies demonstrate that our future vision of cell-triggered surfaces is feasible and warrants further exploration.

## Materials and methods

### Surface synthesis

Surfaces were synthesised using SPPS (Solid phase peptide synthesis) as in Ref.^38^ (Todd et al. ^36^). Briefly, glass coverslips were sonicated in acetone, ethanol, methanol, deionised water (dH_2_O) for 10 min each and allowed to dry in a fume hood. The dried coverslips were acid cleaned in piranha solution (H_2_O_2_: H_2_SO_4_, 3:7) for 1 h. Coverslips were then rinsed in deionised water (dH_2_O) until the solution was neutralised, and coverslips were rinsed individually in 3× dH_2_O before drying with nitrogen. As per Piehler et al*.* surfaces were silanised by immersing the coverslips in GOPTS ((3-glycidyloxypropyl) trimethoxysilane, Sigma Aldrich, UK) at 37 °C for 1 h followed by washing 3× in acetone and drying overnight at 75 °C in an oven. PEG_26_-NH_2_ (Polypure, Norway) was melted onto the silanised surface at 75 °C for 48 h to create an amine-functionalised monolayer. Excess PEG_26_-NH_2_ was removed by washing the surfaces 3× with dH_2_O and dried under nitrogen. To build up the peptide chain, the first Fmoc protected amino acid (20 mM, Sigma Aldrich, UK) was coupled to PEG_26_ diamine in a solution of EHICA (ethyl(hydroxyimino)cyanoacetate, 0.4 mmol) and DIC (*N*,*N*′-diisopropyl-carbodiimide, 0.4 mmol) per 10 mL of anhydrous DMF (*N*,*N*-dimethylformamide) (all reagents from Sigma Aldrich, UK). Samples were treated for 2 h under agitation.

Samples were then rinsed in DMF, ethanol, methanol and DMF for 10 min each under agitation. For the conjugation of subsequent amino acids, the Fmoc protecting group (of the bound amino acid) was removed using piperidine (20% in DMF, Sigma Aldrich, UK) for 2 h under agitation then washed in DMF, ethanol, methanol and DMF for 10 min. The next Fmoc protected amino acid was added and the last two steps were repeated until the sequence was complete. The Fmoc protecting group was removed from the final peptide then PEG (O-methyl-O′-succinyl polyethylene glycol 2.000, Sigma Aldrich, UK) was added to the terminal amino acid prior to removal of side chains. The side chain protecting groups on the aspartic acid (*O-tert-butyl*, OtBu) and the arginine (pentamethyldihydrobenzofuran-5-sulfonyl, Pbf) were removed with a 90% solution of aqueous TFA (trifluoroacetic acid, Sigma Aldrich, UK) for 4 h. Samples were then washed and stored in a desiccator until further use.

### Fluorescent spectroscopy

Coverslips were saved after Fmoc protected amino acid addition and after deprotection by piperidine to indicate all stages of SPPS. Coverslips were dried and mounted onto a microscope slide and fluorescence spectra recorded with a JASCO FP-6500 spectrophotometer using a technique that was specifically developed for SPPS coverslips by Zelzer et al*.*^44^. Coverslips were angled a 30° from the incident light and exposed to an emission spectra = 270 nm and excitation spectra = 320 nm with a slit width of 20 nm (light source and detector).

### Water contact angle measurement

Water contact angle measurements were carried out using the sessile drop technique (3 µL droplets, spotted 5 times per coverslip in triplicate). Angle recording as was conducted using a Theta optical tensiometer (Biolin Scientific, Stockholm Sweden).

### Tof SIMS

ToF–SIMS was carried out using an ION-TOF TOF–SIMS IV instrument (Münster, Germany), equipped with a Bi liquid metal ion gun (LMIG). The primary ion beam was directed at the sample under an angle of 45° in relation to the normal; 25 keV Bi^3+^ primary ions were used in all measurements. Charging of the sample was compensated using low-energetic electrons from the flood gun. Large scale (3 mm × 3 mm; 304 × 304 pixels) and small scale (380 µm × 380 µm, 256 × 256 pixels) images were obtained in positive polarity for each sample (1 shot per pixel). Positive ion mass spectra were calibrated with m/z 15 (CH_3_^+^), 29 (C_2_H_5_^+^), 41 (C_3_H_5_^+^) 67 (C_5_H_7_^+^) and 91 (C_7_H_7_^+^). A peak search was performed to identify ions indicative for amino acids according to data previously reported in the literature (Wagner and Castner^46^). For semi-quantitative comparison of ion intensities, the large-scale images were divided into four regions of interest (ROIs) of 1.5 mm × 1.5 mm from which intensities of ions of interest were generated. Spectra used for peak shape comparison were extracted from the small-scale images.

### Cell culture

STRO-1 Selected SSCs. STRO-1 + SSCs were derived from waste human bone marrow samples obtained from haematologically normal patients undergoing hip replacement surgery at Southampton General Hospital and Spire Southampton Hospital following informed consent. Use of samples was approved by the University of Southampton local ethics committee (NRES number: 194/99/1, LREC number: 31875) and all methods were performed in accordance with the relevant guidelines and regulations. Cells were aspirated from trabecular bone marrow samples and centrifuged at 250×*g* for 4 min at 4 °C. The cell pellet was resuspended inα-MEM and passed through a 70 μm pore nylon mesh (BD Biosciences). Red blood cells were removed by centrifugation with lymphoprep gradient solution (Robbins Scientific) and the remaining cells in the buffy layer resuspended in 10 mL of blocking solution (4-(2-hydroxyethyl)-1-piperazine ethanesulfonic acid, HEPES) saline solution with 5% v/v fetal calf serum, 5% v/v human serum, and 1% w/v bovine serum albumin (BSA). Afterward, the cells were incubated with a STRO-1 antibody in hybridoma supernatant (hybridoma courtesy of Dr Beresford, University of Bath) and flushed with magnetic cell separation buffer (Miltenyi Biotec) to remove any excess antibody. The cells were incubated with human anti-IgM magnetic microbeads (Miltenyi Biotec, UK) and added to a magnetic column; the eluent was collected as the STRO-1− fraction. After being washed with MACs buffer without the magnetic field, the eluted cell population was collected as the STRO-1+ fraction. Skeletal MSCs purchased from Promocell GBMH were used for cytoskeletal staining and focal adhesion experiments.

For all experiments, SSCs were cultured in complete DMEM–DMEM (Sigma-Aldrich, UK) supplemented with 10% FBS (Sigma), 1% sodium pyruvate (11 mg/mL, Sigma), 1% Gibco MEM NEAA (amino acids, Thermo Fisher Scientific, UK) and 2% antibiotics (6.74 U/mL penicillin–streptomycin, 0.2 µg/mL fungizone) and maintained at 37 °C in 5% CO_2_. To detach the SSCs, cells were rinsed in HEPES saline, then incubated with 5 mL of 10% trypsin in versene (37 °C in 5% CO_2_) until detached. 5 mL culture media was added to halt the action of the trypsin, the resulting cell suspension was transferred to into 15 mL falcon tube and centrifuged for 4 min at 1400×*g* to sediment cells.

Prior to cell seeding, functionalised coverslips were incubated in 70% ethanol for 10 min then air dried. After centrifuging, cells were counted using a haemocytometer then seeded at 1000 cells/cm^2^ in 1 mL culture media. Coverslips were incubated for the time stated with media changes performed twice weekly. For MMP experiments, to maintain presence of cell secreted MMPs, 500 µL media was removed from the well and topped up with a fresh 500 µL.

### MMP arrays

Cells were cultured in complete DMEM on coverslips for 3 weeks after which the cell supernatant was pooled (n = 3) and stored at − 80 °C until further use. The experiment was carried out as outlined in the manufacturer’s instructions (Abcam Human MMP Antibody Array), where 1 mL of undiluted pooled sample was added to the membrane and incubated overnight. The protein of interest was captured by antibody array chips (“spots”), biotin-conjugated antibodies and then labelled streptavidin for detection. The membranes were analysed with an Azure c500 Infrared Western Blot Imaging System and analysed using Fiji software (ImageJ derivative, free download from NIH). Pixel density was calculated for each spot and then averaged.

### Zymography

Supernatant was collected from cell culture on functionalised coverslips stored at − 80 °C prior to zymogram analysis. Supernatant was then mixed 1:1 with zymogram sample buffer (loading buffer, BioRad, UK) and 20 µL was loaded into each well of precast gelatin and casein gels (BioRad, UK). Precision Plus Protein ™ Dual Colour Standards (BioRad, UK) were used as molecular standards (10 µL loaded per gel). The gels were run at 200 V in running buffer (10% Tris/Glycine/SDS running buffer diluted PBS) for 60 min in the Criterion™ gels until the bands reached the bottom. Gels were soaked in zymogram renature buffer (10% renature buffer in PBS, BioRad UK) using gentle agitation for 45 min with buffer changes at 15 and 30 min. The gels were then incubated in zymogram development buffer (10% development buffer in PBS) overnight at 37 °C. Afterwards, gels were stained in 0.5% (w/v) Coomassie Brilliant Blue R-250 in 4% methanol 10% acetic acid at room temperature for 60 min with gentle agitation. The Coomassie Blue solution was replaced with destain solution (4% methanol/10% acetic acid in PBS), which was replaced with 3 changes every 15 min (at room temperature) until bands were visible. Gels were imaged using a fusion Fx, Vilber Lourmat and bands were quantified using Fiji software (ImageJ derivative, free download from NIH).

### ELISA

MMP-2 (Life Technologies, UK) and MMP-9 (Invitrogen, UK) ELISAs were carried out as per the manufacturers instruction. For each ELISA, samples of DMEM were included as controls and cell supernatant was diluted 1/10 with standard diluent. For MMP-2 ELISA, an additional positive control was also included of 0.1 mg/mL MMP-2 spiked (Sigma Aldrich, USA) serum free media (complete DMEM without FBS addition). ELISA plates were read at 450 nm using Clariostar microplate reader (BMG Labtech, Germany).

### Viability

#### Live/dead

Cells were stained after 24 h using 1 µL of calcein and 1 µl ethidium homodimer-1 (both Invitrogen, UK), which was added to 1 mL of cell culture media (DMEM). 100 µL of solution was added to controls for 30 min (37 °C, 5% CO_2_). The coverslips were then inverted and placed on microscope slides. Slides were then imaged using a Zeiss Axiophot fluorescence microscope with an Evolution QEi digital monochromatic CCD camera and Q-capture imaging software.

#### MTT

SSCs were cultured for 1 week. Samples were rinsed in 1× PBS. A 5 mg/mL solution of MTT powder (Sigma Aldrich, UK) in 1× PBS was added to the samples 1:10 in DMEM. The cells were placed on a shaking plate for 5 min then incubated under standard conditions (37 °C, 5% CO_2_) for 5 h. The media was removed and the cells were washed twice in cold PBS. 200 µL of dimethyl sulfoxide (DMSO, Sigma Aldrich, UK) was added, then the cells were shaken for 5 min. The solution was transferred to a new 96 well plate and analysed using a Clariostar microplate reader (BMG Labtech, Germany).

#### Alamar blue

SSCs were cultured for 1 week under standard conditions. Alamar blue solution (BioRad, UK) was mixed 1:10 in DMEM then 600 µL was added to each coverslip then incubated for 6 h (30 °C, 5% CO_2_). 3 × 200 µL of solution (supernatant) was transferred to a 96 well plate and analysed using a Thermo-Scientific Multiskan FC microplate reader.

### Cytoskeletal/BrdU staining

SSCs were seeded on surfaces for 24 h Samples were washed with PBS and fixed with 10% v/v formaldehyde/PBS for 15 min at 37 °C. Cells were permeabilized at 4 °C for 5 min (30 mM sucrose, 50 mM NaCl, 3 mM MgCl_2_·6H_2_O, 20 mM HEPES, and 0.5% v/v Triton X-100 in PBS adjusted to pH 7.2), and nonspecific binding epitopes were blocked with 1% w/v BSA/PBS for 15 min at 37 °C (this step was omitted for anti-BrdU staining). Primary antibodies (tubulin, vinculin and vimentin, all mouse monoclonal from Sigma Aldrich UK) were made up at 1:150 dilution in PBS/BSA with rhodamine –phalloidin (1:500; Molecular Probes) and incubated for 1 h at 37 °C, after which time they were washed in 0.5% v/v Tween 20/PBS (PBST, 3 × 5 min under gentle agitation) to minimize background labelling. Note that for BrdU staining, mouse monoclonal anti-BrdU (clone BU-1, 1:100 in nuclease solution, prepared according to the manufacturer’s instructionsin kit RPN202; GE Healthcare) was used and rhodamine − phalloidin was added with the secondary rather than the primary antibody.

Horse biotinylated anti-mouse IgG (1:50; Vector Laboratories) in BSA/PBS was added to samples and incubated for 1 h at 37 °C. After the washing stages, samples were incubated for 30 min at 4 °C with fluoresceinisothiocyanate streptavidin (FITC; 1:50; Vector Laboratories) in BSA/PBS followed by a final washing stage. Coverslips were placed on glass slides in 4′,6-diamidino-2-phenylindole mountant (Vector Laboratories). Images taken using a Zeiss Axiophot fluorescence microscope with an Evolution QEi digital monochromatic CCD camera and Q-capture imaging software.

### In cell western (ICW) analysis

Supernatant was removed from the wells and samples were rinsed in PBS. Cells were then fixed with 10% v/v formaldehyde/PBS for 15 min at 37 °C and permeabilised at 4 °C for 4 min. Permeablisation buffer (30 mM sucrose, 50 mM NaCl, 3 mM MgCl_2_·6H_2_O, 20 mM HEPES, and 0.5% v/v Triton X-100 in PBS adjusted to pH 7.2) was removed and milk protein (1% milk protein in 1× PBS) was added at 37 °C for 1.5 h on a shaker after which, stro-1, ALP, OCN and OPN primary antibodies (mouse monoclonal, 1/100, Santa Cruz, UK) in PBS/BSA was added and incubated at 37 °C for 2.5 h. Samples were washed 5 × 5 min in tween, then the secondary antibody with a 680 nm infrared dye (1/800) and CellTag™ (1/500) (both diluted in milk protein, both Licor, UK) were added for 1 h at room temperature. Washing was carried out on a shaker 5× in tween for 5 min each at room temperature. Coverslips were removed and dried on white paper then inverted into a new 24 well plate. ICW was performed using a LI-COR Odyssey plate reader and data was analysed using Odyssey SA software.

### Quantitative PCR

RNA was extracted using the Qiagen RNeasy extraction kit (including a DNAse step) according to the manufacturers instructions (Qiagen, Hilden, Germany). The RNA concentration was quantified using Nanodrop, and normalized for each sample. cDNA was prepared by reverse transcription using the Qiagen Quantitect kit. 12 µL of the normalised dilutions for each RNA sample were added to a 0.2 mL (200 µL) reverse transcription PCR–Cup. To each of the samples 2 µL gDNA wipeout buffer was added. Following this each sample was run at 42 °C for 2 min, on a thermal cycler in order to remove any DNA present in the samples. Following the removal of the innate DNA, 6 µL of a stock solution (containing Qiagen reverse transcription buffer reagents) was then added to each reverse transcription cup, to give a total volume of 20 µL. Reverse transcription of the RNA into cDNA was performed by controlling temperature from 42 (15 min) to 93 (3 min) to 4 °C (hold) to allow PCR quantification using the relative comparable method. The Quantifast SYBR green qPCR kit (Qiagen) was used to perform amplification with specific primers (Table 2, Eurofins Genomics, Ebersberg, Germany) related to osteogenesis as well as GAPDH as a genetic internal control. PCR was quantified using the 2^−∆∆Ct^ method and amplification was carried out using an Applied Biosystems 7500 Real Time PCR system.

**Table 2:** PCR primers.

Target	Primer	
CD63	Back	5′-ATCCCACAGCCCACAGTAAC-3′
	Forward	5′-GCTGTGGGGCTGCTAACTAC-3′
Sox9	Back	5′-CGGCAGGTACTGGTCAAACT-3′
	Forward	5′-AGACAGCCCCCTATCGACTT-3′
PPARγ	Back	5′-CTGCAGTAGCTGCACGTGTT-3′
	Forward	5′-TGTGAAGCCCATTGAAGACA-3′
Col 1a	Back	5′-AGGTGAAGCGGCTGTTGCC-3′
	Forward	5′-GCTCCGACCCTGCCGATGTG-3′
GAPDH	Back	5′-TGGGTGGCAGTGATGGCA-3′
	Forward	5′-TCAAGGCTGAGAACGGGAA-3′

## Supplementary Information

Supplementary Figures.

## References

[1] Mirmalek-Sani SH (2006). Characterization and multipotentiality of human fetal femur-derived cells—Implications for skeletal tissue regeneration. Stem Cells.

[2] Bianco P, Riminucci M, Gronthos S, Robey PG (2001). Bone marrow stromal stem cells: Nature, biology, and potential applications. Stem Cells.

[3] Tome M, Lindsay SL, Riddell JS, Barnett SC (2009). Identification of nonepithelial multipotent cells in the embryonic olfactory mucosa. Stem Cells.

[4] Watt FM, Hogan BL (2000). Out of Eden: Stem cells and their niches. Science.

[5] Yang C, Tibbitt MW, Basta L, Anseth KS (2014). Mechanical memory and dosing influence stem cell fate. Nat. Mater..

[6] McMurray RJ (2011). Nanoscale surfaces for the long-term maintenance of mesenchymal stem cell phenotype and multipotency. Nat. Mater..

[7] Tsimbouri PM (2012). Using nanotopography and metabolomics to identify biochemical effectors of multipotency. ACS Nano.

[8] Lee LC (2017). Nanotopography controls cell cycle changes involved with skeletal stem cell self-renewal and multipotency. Biomaterials.

[9] Dalby MJ, Garcia AJ, Salmeron-Sanchez M (2018). Receptor control in mesenchymal stem cell engineering. Nat. Rev. Mater..

[10] Qiryaqoz Z, Timilsina S, Czarnowski D, Krebsbach PH, Villa-Diaz LG (2019). Identification of biomarkers indicative of functional skeletal stem cells. Orthod. Craniofac. Res..

[11] Rayagiri SS (2018). Basal lamina remodeling at the skeletal muscle stem cell niche mediates stem cell self-renewal. Nat. Commun..

[12] Docheva D, Popov C, Alberton P, Aszodi A (2014). Integrin signaling in skeletal development and function. Birth Defects Res. C Embryo Today.

[13] McBeath R, Pirone DM, Nelson CM, Bhadriraju K, Chen CS (2004). Cell shape, cytoskeletal tension, and RhoA regulate stem cell lineage commitment. Dev. Cell.

[14] Kilian KA, Bugarija B, Lahn BT, Mrksich M (2010). Geometric cues for directing the differentiation of mesenchymal stem cells. Proc. Natl. Acad. Sci. U.S.A..

[15] Lee J, Abdeen AA, Tang X, Saif TA, Kilian KA (2015). Geometric guidance of integrin mediated traction stress during stem cell differentiation. Biomaterials.

[16] Friedenstein AJ (1976). Precursor cells of mechanocytes. Int. Rev. Cytol..

[17] Monroe DG, Hawse JR, Subramaniam M, Spelsberg TC (2010). Retinoblastoma binding protein-1 (RBP1) is a Runx2 coactivator and promotes osteoblastic differentiation. BMC Musculoskelet. Disord..

[18] Ogasawara T (2004). Bone morphogenetic protein 2-induced osteoblast differentiation requires smad-mediated down-regulation of Cdk6. Mol. Cell. Biol..

[19] Stein GS, Lian JB (1993). Molecular mechanisms mediating proliferation/differentiation interrelationships during progressive development of the osteoblast phenotype. Endocr. Rev..

[20] Yang J (2014). Nanotopographical induction of osteogenesis through adhesion, bone morphogenic protein cosignaling, and regulation of MicroRNAs. ACS Nano.

[21] Giannoudis PV, Chris Arts JJ, Schmidmaier G, Larsson S (2011). What should be the characteristics of the ideal bone graft substitute?. Injury.

[22] Dimitriou R, Mataliotakis GI, Angoules AG, Kanakaris NK, Giannoudis PV (2011). Complications following autologous bone graft harvesting from the iliac crest and using the RIA: A systematic review. Injury.

[23] Garcia-Gareta E, Coathup MJ, Blunn GW (2015). Osteoinduction of bone grafting materials for bone repair and regeneration. Bone.

[24] Tsimbouri PM (2017). Stimulation of 3D osteogenesis by mesenchymal stem cells using a nanovibrational bioreactor. Nat. Biomed. Eng..

[25] Ho-Shui-Ling A (2018). Bone regeneration strategies: Engineered scaffolds, bioactive molecules and stem cells current stage and future perspectives. Biomaterials.

[26] Childs PG, Reid S, Salmeron-Sanchez M, Dalby MJ (2020). Hurdles to uptake of mesenchymal stem cells and their progenitors in therapeutic products. Biochem. J..

[27] Celiz AD (2014). Materials for stem cell factories of the future. Nat. Mater..

[28] DeForest CA, Tirrell DA (2015). A photoreversible protein-patterning approach for guiding stem cell fate in three-dimensional gels. Nat. Mater..

[29] Yeo WS, Mrksich M (2004). Electroactive substrates that reveal aldehyde groups for bio-immobilization. Adv. Mater..

[30] Mendes PM (2013). Cellular nanotechnology: Making biological interfaces smarter. Chem. Soc. Rev..

[31] Weis S, Lee TT, del Campo A, Garcia AJ (2013). Dynamic cell-adhesive microenvironments and their effect on myogenic differentiation. Acta Biomater..

[32] Maitz MF (2013). Bio-responsive polymer hydrogels homeostatically regulate blood coagulation. Nat. Commun..

[33] Khetan S (2013). Degradation-mediated cellular traction directs stem cell fate in covalently crosslinked three-dimensional hydrogels. Nat. Mater..

[34] Chaudhuri O (2016). Hydrogels with tunable stress relaxation regulate stem cell fate and activity. Nat. Mater..

[35] Zelzer M, Todd SJ, Hirst AR, McDonald TO, Ulijn RV (2013). Enzyme responsive materials: Design strategies and future developments. Biomater. Sci..

[36] Biggs MJ (2009). The use of nanoscale topography to modulate the dynamics of adhesion formation in primary osteoblasts and ERK/MAPK signalling in STRO-1+ enriched skeletal stem cells. Biomaterials.

[37] Todd SJ (2009). Enzyme-activated RGD ligands on functionalized poly(ethylene glycol) monolayers: surface analysis and cellular response. Langmuir.

[38] Roberts JN (2016). Dynamic surfaces for the study of mesenchymal stem cell growth through adhesion regulation. ACS Nano.

[39] Silva WA (2003). The profile of gene expression of human marrow mesenchymal stem cells. Stem Cells.

[40] Howard D (2002). Immunoselection and adenoviral genetic modulation of human osteoprogenitors: In vivo bone formation on PLA scaffold. Biochem. Biophys. Res. Commun..

[41] Kanczler J (2019). Isolation, differentiation, and characterization of human bone marrow stem cells in vitro and in vivo. Methods Mol. Biol..

[42] Huang W (2012). Mesenchymal stem cells overexpressing CXCR4 attenuate remodeling of postmyocardial infarction by releasing matrix metalloproteinase-9. Stem Cells Dev..

[43] Rawlings ND, Barrett AJ, Bateman A (2010). MEROPS: The peptidase database. Nucleic Acids Res..

[44] Zelzer M, Scurr DJ, Alexander MR, Ulijn RV (2012). Development and validation of a fluorescence method to follow the build-up of short peptide sequences on solid 2D surfaces. ACS Appl. Mater. Interfaces.

[45] Kilian KA, Mrksich M (2012). Directing stem cell fate by controlling the affinity and density of ligand-receptor interactions at the biomaterials interface. Angew. Chem. Int. Ed..

[46] Wagner MS, Castner DG (2001). Characterization of adsorbed protein films by time-of-flight secondary ion mass spectrometry with principal component analysis. Langmuir.

