# Supplementary material for: Cell-controlled dynamic surfaces for skeletal stem cell growth and differentiation
Source: Sci Rep. 2022 May 17;12:8165. doi: 10.1038/s41598-022-12057-z (PMC9114122; doi:10.1038/s41598-022-12057-z)
Supplement: Supplementary file 1 — Supplementary Figures. [file 41598_2022_12057_MOESM1_ESM.docx]

Supplementary data for:

**Cell-controlled switchable surfaces for skeletal stem cell growth and differentiation.**

Hilary J Anderson, Jugal Kishore Sahoo, Julia Wells, Sebastiaan van Nuffel, Hala S Dhowre, Richard OC Oreffo, Mischa Zelzer, Rein V Ulijn & Matthew J Dalby.


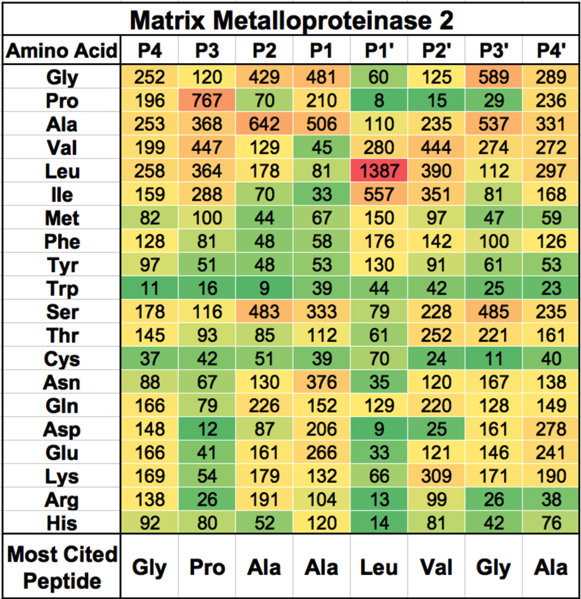


***Supplementary figure 1.*** *MEROPS data on citations to positions around the sessile bond cleaved by MMP-2.*

*
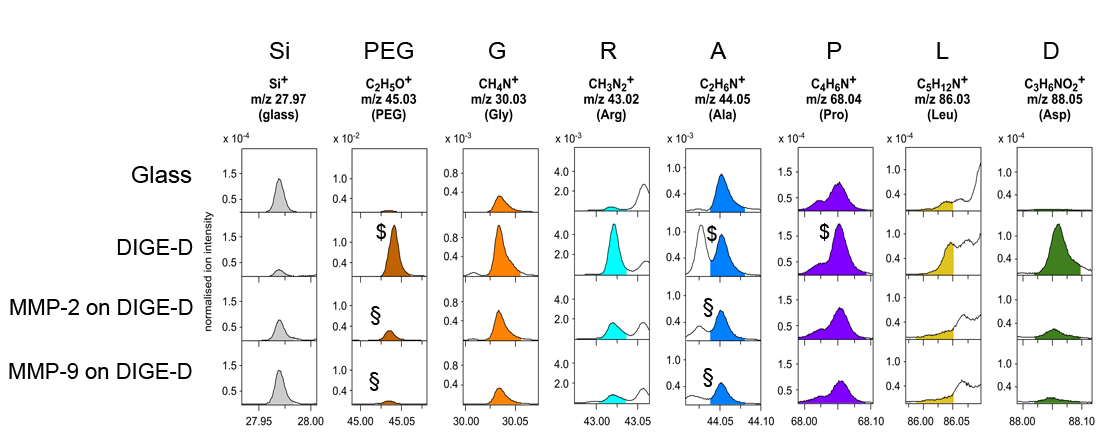
*

***Supplementary figure 2.*** *Tof-SIMS data showing that all amino acids are present on the complete, DIGE-D, surface. After treatment with either 20 ng/ml of active MMP-2 or 0.25 ng/ml of active MMP-9 in serum free media, the intensity of the ion associated with PEG (C_2_H_5_O^+^) is reduced while that of the substrate (Si^+^) increases, indicating removal of PEG from the surface.*

*
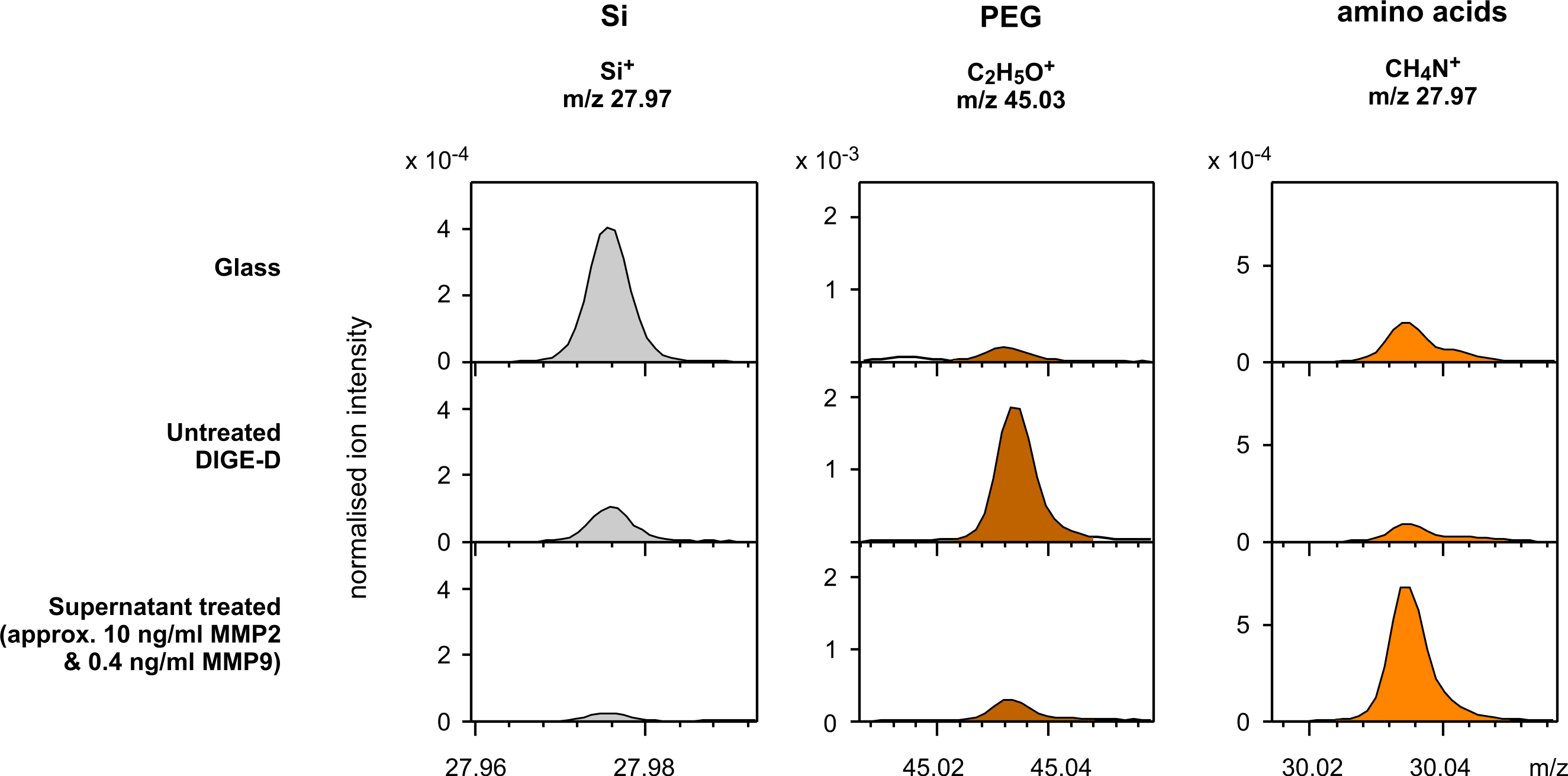
*

***Supplementary figure 3.*** *Tof-SIMS data showing that at the same time as decrease in PEG signal with addition of MMPs, that there is increased protein (CH_4_N^+^) signal detected.*

*
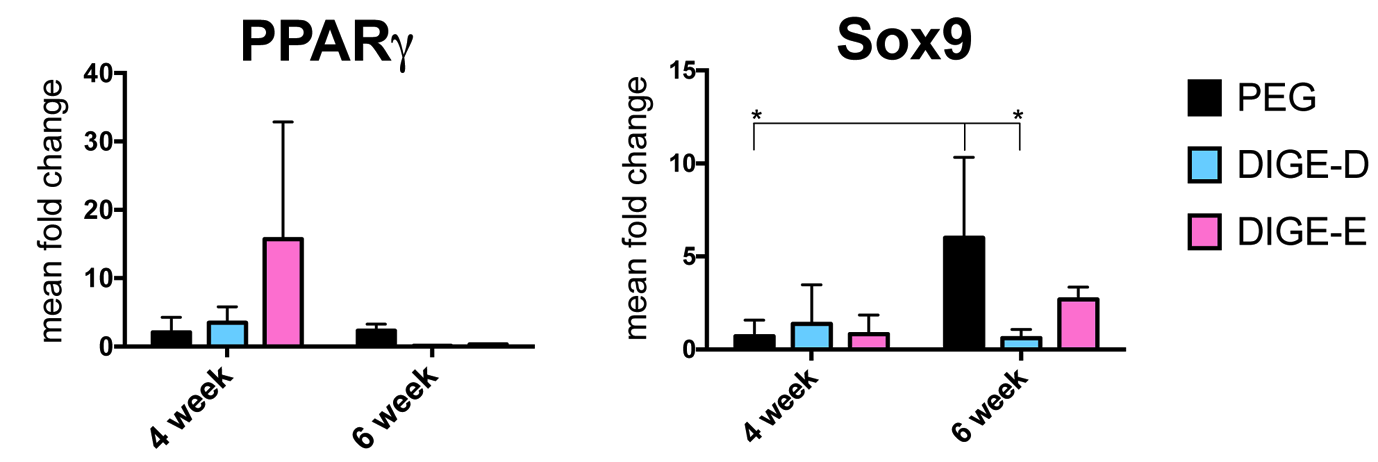
*

***Supplementary figure 4.*** *QPCR for PPARGγ and SOX9 transcripts after 4 and 6 weeks of MSC culture on PEG, LRGD and DIGE-D surfaces. No sign of adipogenesis was seen at either 4 or 6 weeks. There was some evidence of potential chondrogenesis on the PEG controls but not the DIGE-D or DIGE-E surfaces. Graphs show mean ± SD, n=3, statistics by ANOVA and Tukey test where *=p<0.05, **=p<0.01, ***=p<0.001 and ****=p<0.0001. If stars are not shown on the graphs, it denotes no significant difference was observed between the treatment and relevant control.*
